# Atypical Femoral Shaft Fractures in Female Bisphosphonate Users Were Associated with an Increased Anterolateral Femoral Bow and a Thicker Lateral Cortex: A Case-Control Study

**DOI:** 10.1155/2017/5932496

**Published:** 2017-03-28

**Authors:** Seung Pil Jang, Ingwon Yeo, Sang-Yeon So, Keunbyuel Kim, Young-Wan Moon, Youn-Soo Park, Seung-Jae Lim

**Affiliations:** ^1^Graduate School of Medical Science and Engineering, Korea Advanced Institute of Science and Technology, Daejeon, Republic of Korea; ^2^Samsung Medical Center, Sungkyunkwan University School of Medicine, Seoul, Republic of Korea

## Abstract

The purpose of our study was to investigate the radiographic characteristics of atypical femoral shaft fractures (AFSFs) in females with a particular focus on femoral bow and cortical thickness. We performed a fracture location-, age-, gender-, and ethnicity-matched case-control study. Forty-two AFSFs in 29 patients and 22 typical osteoporotic femoral shaft fractures in 22 patients were enrolled in AFSF group and control group, respectively. With comparing demographics between two groups, radiographically measured femoral bow and cortical thicknesses of AFSF group were compared with control group. All AFSF patients were females with a mean age of 74.4 years (range, 58–85 years). All had a history of bisphosphonate (BP) use with a mean duration of 7.3 years (range 1–17 years). Femoral bow of AFSF group was significantly higher than control group on both anteroposterior (AP) and lateral radiographs after age correction. Mean femoral bow on an AP radiograph was 12.39° ± 5.38° in AFSF group and 3.97 ± 3.62° in control group (*P* < 0.0001). Mean femoral bow on the lateral radiograph was 15.71° ± 5.62° in AFSF group and 10.72° ± 4.61° in control group (after age correction *P* = 0.003). And cortical thicknesses of AFSF group demonstrated marked disparity between tensile and compressive side of bowed femurs in this study. An adjusted lateral cortical thickness was 10.5 ± 1.4 mm in AFSF group and 8.1 ± 1.3 mm in control group (after age correction *P* < 0.0001) while medial cortical thickness of AFSF group was not statistically different from control group. Correlation analysis showed that the lateral femoral bow on the AP radiograph was solely related to lateral CTI (*R* = 0.378, *P* = 0.002). AFSFs in female BP users were associated with an increased anterolateral femoral bow and a thicker lateral cortex of femurs.

## 1. Introduction

Bisphosphonate (BP) is widely used for the treatment of osteoporosis, Paget's disease, metastatic bone disease, multiple myeloma, and hypercalcemia of malignancy. Particularly in the field of osteoporosis, BPs have become the most clinically important class of antiresorptive medications [1]. Oral BPs (alendronate, risedronate, and ibandronate), mainly used for the treatment of osteoporosis, have been associated with adverse events from the upper gastrointestinal tract, acute phase response, hypocalcemia and secondary hyperparathyroidism, musculoskeletal pain, osteonecrosis of the jaw, and ocular events. Among them, atypical femoral fracture (AFF) has recently emerged as one of the most concerning issues while managing osteoporosis.

According to previous radiographic adjudication studies and large database studies, the incidence of AFFs can be estimated between 0.3 and 11 per 100,000 person years [2–5]. Despite its low incidence rate of AFF, considerable concern about AFF exists among physicians and patients regarding long-term use of BP for fracture prevention.

For differentiation of AFFs from typical osteoporotic femoral fractures, the ASBMR task force suggested the diagnostic criteria of the 2014-revised edition of the ASBMR [6]. For example, the fracture must be along the femoral diaphysis from just distal to the lesser trochanter to just proximal to the supracondylar flare, and at least four of the five major features must be present. The major features of AFF are as follows: (1) the fracture is associated with minimal or no trauma (e.g., a fall from a standing height or less); (2) the fracture line originates at the lateral cortex and is substantially transverse, although it may become oblique as it progresses medially across the femur; (3) complete fractures extend through both cortices and may be associated with a medial spike (incomplete fractures involve only the lateral cortex); (4) the fracture is noncomminuted or minimally comminuted; (5) and localized periosteal or endosteal thickening of the lateral cortex is present at the fracture site (i.e., beaking or flaring).

It is notable that AFFs also occur in BP-naive individuals and Asian race is an increasingly recognized risk factor for AFFs [7, 8], suggesting that the pathophysiology of AFFs cannot be entirely explained by BPs. Previously, specific femoral geometries such as varus neck shaft angles or shorter limbs have been identified to play a role in AFF pathogenesis [9, 10]. Recently, Morin et al. documented that femoral bow was more prevalent in patients with AFSF, also the most common site of AFF in the same vein [11]. Some reports on stress fractures of the femoral shaft after total knee arthroplasty have also described involvement of femoral shaft bowing deformity and bilaterality [12, 13]. Additionally the presence of a lateral cortical thickening in these fractures was earlier described by Kwek et al. [14], and this localized periosteal reaction of the lateral cortex has been classified by the ASBMR task force report as a minor feature of AFFs [15]. More importantly, lower limb geometry has emerged as possible predisposing factors for AFFs by the Task Force of the ASBMR in 2013 [6].

However, there is still a lack of scientific researches on the femoral geometries potentially responsible for AFSF formation [6], which has attracted our interest. The primary objective of this study was to investigate the radiographic characteristics of atypical femoral shaft fracture (AFSF) in postmenopausal female patients, particularly focusing on femoral bow and cortical thickness. And the correlation between femoral bow and cortical thicknesses of AFSFs in this study population was also investigated.

## 2. Methods

### 2.1. Study Population

After Institutional Review Board approval (SMC IRB number 2014-11-089), from chart review, we used a case-control design to determine if women with a BPs-associated AFSF have different femoral geometrical parameters than fracture location-, age-, gender-, and ethnicity-matched controls with typical femoral shaft fracture. AFSF group consisted of female patients aged 50 or over referred to Samsung Medical Center, Seoul, Korea, and had been diagnosed with BPs-associated AFSF between June 1st, 2009, and January 31st, 2017. In our study, the strict set of clinical and radiological criteria to define AFSFs was important to differentiate them from TFSF. It was assumed that patients with AFSFs fulfilled all the diagnostic criteria of the 2014-revised edition of the ASBMR as mentioned earlier [6]. Control group consisted of female patients aged 50 or over referred to the same facility and had been diagnosed with TFSF at the same periods. For BPs therapy, a continuous BP treatment interval was calculated excluding all treatment gaps.

Any patients with a previous history of (1) any ipsilateral femoral fracture (irrespective of the site of previous fracture), (2) periprosthetic fractures, (3) bilateral femoral shaft complete fracture with angular deformity, (4) subtrochanteric fractures, (5) pathologic fractures, or (6) comorbidities such as rheumatoid arthritis, systemic glucocorticoid exposure within 1 year (more than 5 mg/day), and other disorders requiring proton pump inhibitors or aromatase inhibitors within 1 year were excluded from this study. If the patient had sustained simultaneous bilateral AFSFs, with at least one incomplete fracture without any angular deformity, the patient was included in analysis. After reviewing radiographs and electronic medical records, 42 femurs (29 patients) and 22 femurs (22 patients) were enrolled as AFSF group and control group, respectively, in this study.

### 2.2. Measurements and Definitions

In the absence of a specific ICD code for AFSF, the femoral radiographs of all the patients listed in the hospital's administrative database with a code for subtrochanteric or diaphyseal femur fracture between June 1st, 2009, and January 31st, 2017, were reviewed. The radiographs were reviewed by two orthopedic specialists. Femoral shaft fracture is defined by location distal to 5 cm below the lesser trochanter and up to but not including the distal metaphyseal flare. For all suspected AFSFs, the medical records were reviewed to confirm the low-velocity character of the trauma. Age, gender, BP use, mechanism of injury, fracture pattern, bilaterality, presence of prodromal symptom, femoral bow, and medial and lateral cortical thickness were evaluated by reviewing radiographs and electronic medical records.

**Figure 1:**
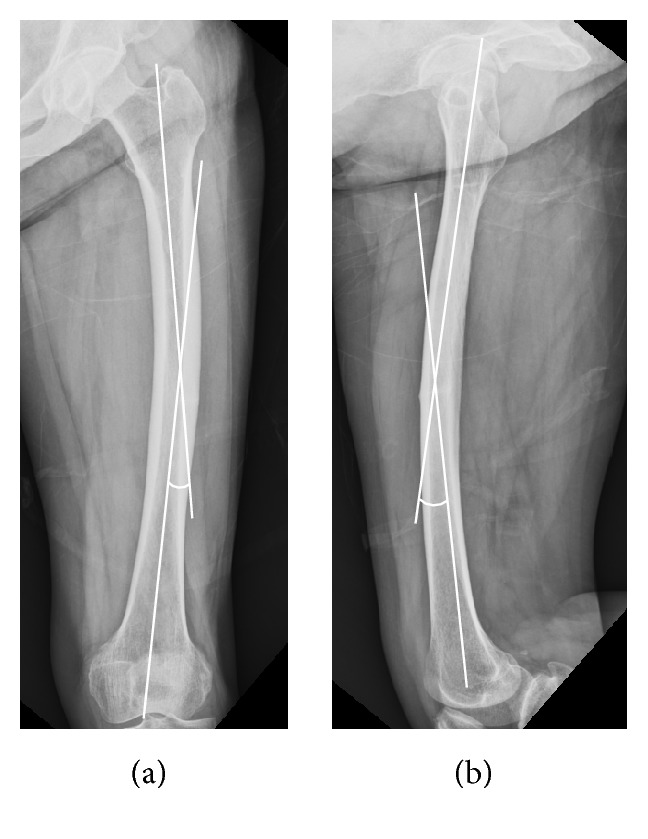
Femoral bows were obtained on both AP (a) and lateral (b) views of contralateral femur. Femoral bows were defined as the angle formed by two lines which are parallel to the proximal and distal portions of femoral shaft.

The femoral bow and medial and lateral cortical thickness of AFSF group were compared with control group. Unlike previous studies [3, 16–20], femoral bow and cortical thickness were measured simultaneously in this study. Femoral bow was defined as the angle formed by two lines parallel to the proximal and distal portions of the femoral shaft, respectively. Femoral bows were obtained from both anteroposterior (AP) and lateral views (Figure 1). A positive value of femoral bow means lateral angulation on the AP view and anterior angulation on the lateral view. A negative value of femoral bow means medial angulation on the AP view and posterior angulation on the lateral view. Femoral bow was measured at the ipsilateral femur in cases of incomplete fracture but was measured at the contralateral femur in cases of complete fracture as we were not able to obtain the radiographs of the femur prior to occurrence of fractures in most cases in both the study and control groups. If the patient had sustained simultaneous bilateral AFSFs with at least one incomplete fracture without angular deformity, femoral bow was measured at the incompletely fractured side.

**Figure 2:**
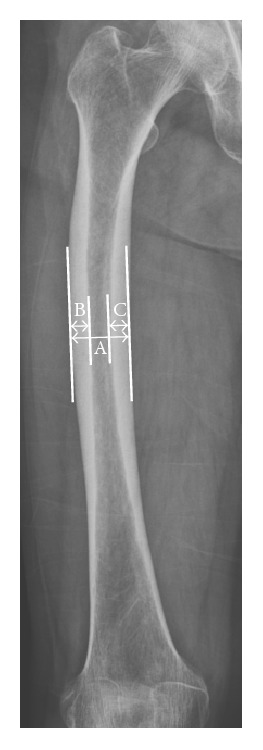
The outer diameter (A) and lateral (B) and medial (C) cortical thickness were measured at the thickest portion of lateral femoral cortex of the femur. Lateral CTI = B/A, medial CTI = C/A, and total CTI = (B + C)/A.

When measuring cortical thickness, we determined the approximate magnification in femoral radiographs performed at our institution with a radiopaque ruler placed horizontally at the level of the greater trochanter. For each case, the magnified ruler distance was measured, which was divided by the true ruler distance to obtain the magnification factor. The magnification was then averaged by two independent observers to obtain the estimated magnification for each femoral radiograph. During measurement, cortical thickening in AFSFs could be mixed up with focal thickening at the fracture which is general phenomenon. In our observation, the medial and lateral cortical thicknesses were measured separately at the thickest portion of the lateral femoral cortex, except at the site where focal periosteal and/or endosteal reactions had occurred (Figure 2). The medial and lateral cortical thicknesses were measured at the ipsilateral femur.

We used the cortical thickness index (CTI) as a standardization method for cortical thickness. This unitless index accounts for differences in radiographic magnification [21] and varying femoral size. CTI was defined as the ratio of the sum of the medial and lateral cortical thicknesses to the outer cortical femoral diameter, measured on a level with the thickest portion of the lateral femoral cortex. Medial CTI was defined as the ratio of the medial cortical thickness to the outer cortical femoral diameter, measured on a level with the thickest portion of the lateral femoral cortex. Lateral CTI was defined as the ratio of the lateral cortical thickness to the outer cortical femoral diameter, measured on a level with the thickest portion of the lateral femoral cortex. Higher values of CTI, medial CTI, and lateral CTI indicate a thicker cortex and vice versa. Adjusted lateral cortical thickness was calculated as the lateral CTI multiplied by the mean femoral diameter of the study group and the control group.

The radiographic analyses and femoral measurements were performed by two independent investigators using standardized techniques on two-dimensional radiographs viewed on the Picture Archiving and Communication System (PACS) software. Each investigator evaluated the radiographs twice, at an interval of 4 weeks (times 1 and 2).

### 2.3. Statistical Analysis

To compare the value of each parameter between the two fracture groups, descriptive statistics were used to determine group means and standard deviations (SD) for numerical data. Differences in the femoral bow, CTI, medial CTI, and lateral CTI were tested for statistical significance using an independent* t*-test. Additionally, we estimated the odds ratio (OR; the relative increase of the prevalence of atypical fracture risk for a variation of 1 SD) of each variable (femoral curvatures, cortical thickness, CTI, medial CTI, and adjusted lateral CTI) using logistic regression analysis in which the OR of each variable was corrected for demographical variables (age and BMI). And, then, we used correlation analysis with age and analysis of covariance (ANCOVA) for correction of age with femoral bow, CTI, medial CTI, and lateral CTI as dependent variables and AFSF as an independent variable and age as a covariate. The relationship between lateral femoral bow and media/lateral CTI of the femur was analyzed by correlation analysis, using Pearson's correlation coefficient (*R*). In addition, we used multiple regression analysis for age correction with lateral femoral bow and age as independent variables and medial/lateral CTI as dependent variables.

Intraobserver and interobserver reliability were also evaluated by calculating the intraclass correlation coefficient (ICC) [22]. ICCs were calculated using two-way mixed mode, absolute agreement, and single measure. Reliability was defined as either excellent (>0.75), fair to good (0.4–0.75), or poor (>0.4). All ICCs for intraobserver and interobserver showed excellent reliability in all measurements.

Statistical analysis was performed using SPSS version 23.0 (IBM Corp., Armonk, NY). Statistical significance was defined as a *P* value < 0.05.

## 3. Results

### 3.1. Baseline Characteristics of Study Population

The demographic parameters were comparable between two groups. All AFSF patients were female and all had a history of BP use with a mean duration of 7.3 years (range 1–17 years). Mean age at the time of fracture was 74.4 years (range, 58–85 years) for AFSF group and 70.9 years (range, 54–84 years) for control group. The BMI in the patients with AFSF was not significantly different from control patients (*P* = 0.910). Of the 42 AFSFs, 15 (36%) had a history of low-energy trauma (fall from standing height or less), whereas the remaining 27 had no history of trauma. Prodromal thigh pain, which was typically located in the anterior and lateral aspect of the midthigh, was present in 24 (57%) out of the 42 femurs. 14 (48%) out of 29 patients had bilateral femoral atypical fracture (one with a contralateral subtrochanteric femur fracture and 13 with contralateral femur shaft fractures). Radiographic analyses revealed that AFSF group had transverse fractures with medial spikes (15 femurs), transverse fractures without medial spikes (4 femurs), short oblique fracture (3 femurs), and incomplete fractures that were limited to lateral cortices (20 femurs).

### 3.2. Radiographic Difference of Femoral Bow and Cortical Thickness between AFSF and TFSF

Of the measured femoral geometry parameters, there were significant differences in femoral bow (both coronally and sagittally), lateral CTI, and total CTI between two groups. Mean femoral bow measured from AP radiograph was 12.39°  ± 5.38° in AFSF group and 3.97°  ± 3.62° in control group (*P* < 0.0001, after age correction *P* < 0.0001). Mean femoral bow on the lateral radiograph was 15.71°  ± 5.62° in AFSF group and 10.72°  ± 4.61° in control group (*P* = 0.002, after age correction *P* = 0.003). We found that femoral bows of AFSF group were significantly higher than control group on both AP and lateral radiographs, regardless of age correction.

The mean lateral CTI of AFSF group (0.323 ± 0.042) was significantly higher than control group (0.267 ± 0.041) (*P* < 0.0001, after age correction *P* < 0.0001). The mean CTI of AFSF group (0.558 ± 0.068) was significantly higher than control group (0.507 ± 0.081) (*P* = 0.009, after age correction *P* = 0.004). The adjusted lateral cortical thickness was 10.5 ± 1.4 mm in AFSF group and 8.1 ± 1.3 mm in control group (*P* < 0.0001, after age correction *P* < 0.0001). However, the mean medial CTI of AFSF group was not statistically different from control group (0.236 ± 0.061 and 0.240 ± 0.048, resp., *P* = 0.773, after age correction *P* = 0.958). A comparison of the radiographic measurement data is summarized in Table 1.

### 3.3. Risks of Atypical Radiologic Features according to Radiographic Parameters

Logistic regression showed that when corrected for demographical variables, the prevalence of atypical fracture increased 1.546-fold (*P* < 0.0001) with a 1-SD increase in the lateral (coronal) femoral bow while it increased 1.220-fold (*P* = 0.005) with a 1-SD increase in the anterior (sagittal) femoral bow in patients with femoral shaft fractures. The prevalence of atypical fracture also increased with a 1-SD increase in the total cortical thickness, lateral cortical thickness, and adjusted lateral cortical thickness by 1.411-fold (*P* = 0.002), 3.234-fold (*P* < 0.0001), and 4.187-fold (*P* < 0.0001), respectively. However, medial cortical thickness and adjusted medical cortical thickness were not statistically related to the risk of atypical fracture in postmenopausal female femoral shaft fractures (Table 2).

### 3.4. Correlation Analysis between Age and Radiographic Parameters

**Figure 3:**
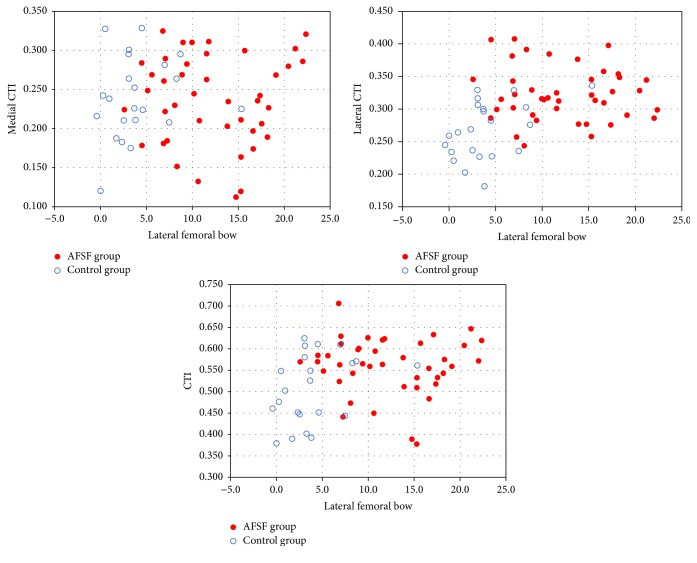
Correlation of medial, lateral, and total CTIs with lateral femoral bow in AFSF group (red filled dot) and control group (blue open rings). The scatter plot shows significant correlation between lateral and total CTI, with lateral femoral bow (*R* = 0.378, *P* = 0.002; *R* = 0.268, *P* = 0.032, resp.). There was no correlation found between medial CTI and lateral femoral bow (*R* = 0.027, *P* = 0.835).

**Figure 4:**
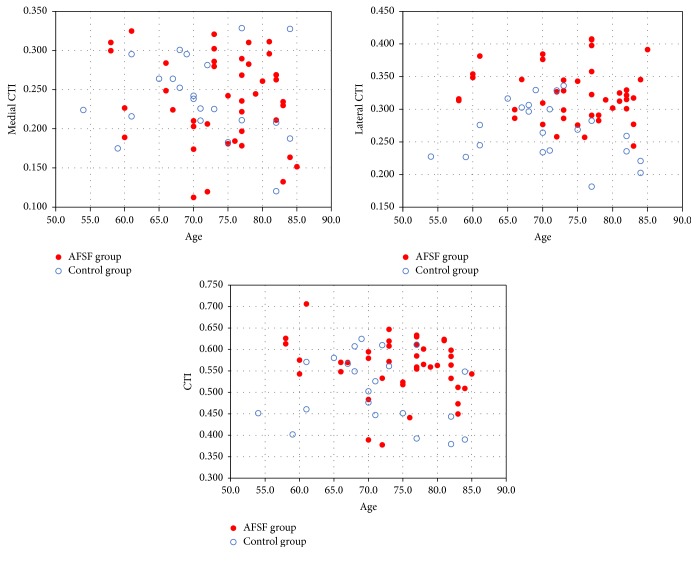
Correlation of medial/lateral/total CTI with age in AFSF group (red filled dot) and control group (blue open rings). The scatter plot shows no correlation between medial, lateral, and total CTIs and age (*P* = 0.276, *P* = 0.967, and *P* = 0.413, resp.).

Our correlation analysis showed that the lateral femoral bow (femur bow on the AP radiograph) was solely related to lateral CTI (*R* = 0.378, *P* = 0.002) (Figure 3). However, medial CTI did not significantly correlate with the lateral femoral bow (*R* = 0.027, *P* = 0.835). And no parameters among femoral bow, CTI, medial CTI, and lateral CTI showed a statistically significant linear relationship with age (Figure 4). Although 2 previous reports from different groups reported that medial and total cortical thickness of femurs decrease with age, age correction may not affect the statistical significance in this present study [3, 16].

## 4. Discussion

In this retrospective case-control study, we evaluated femoral bow and femoral shaft cortical thicknesses at the thickest portion of lateral femoral cortices in 29 postmenopausal BPs-associated AFSF patients (42 femurs), comparing them with control group of 22 TFSF patients (22 femurs) as well.

We found that femoral bows of AFSF group were significantly higher than control group on both AP and lateral radiographs, regardless of age correction. So far, only a few studies have investigated femoral bow in AFSFs. Sasaki et al. measured the femoral bow of 9 patients (12 femurs) who had sustained low-energy femoral shaft fractures and found that the femoral bows of the study group were significantly larger than their control group [17]. But the study was a small case series. Recently, Saita et al. reported that patients with AFSFs had shown an increased standing femorotibial angle (i.e., increased varus of the lower limb) compared to patients with subtrochanteric AFFs or ordinary femoral shaft fractures [23]. Although the study was a case-control study comparing AFFs with TFSF, the number of the case group was only 13 and the fracture locations were not the same in the case group (i.e., 7 subtrochanteric and 6 diaphyseal regions). Morin et al. also found that femoral bow was more prominent in diaphyseal fractures compared to subtrochanteric fractures (−4.3 ± 3.2° versus −0.9 ± 2.7°, *P* = 0.07) in their case-control study regarding AFFs [11]. However, Morin's fracture location-unmatched study was not sufficient to demonstrate a significant relationship between femoral bow and AFFs.

Our result is in agreement with the result of Hyodo et al. who identified a significant relationship between coronal femoral bow and locations of AFFs, which noted that the fracture location was middle diaphysis in all the cases in the presence of coronal femoral bow [24]. Soh et al. and Chen et al. also reported that anterior and lateral femoral bow had linear correlation with the fracture location [25, 26]. According to their data, more distal diaphyseal fractures were observed with larger femoral bow angle compared to atypical subtrochanteric fracture. Possible explanation for an increased femoral bow of AFSF patients is the biomechanics affecting bowed femurs. Medial and posterior shift of the load axis by femoral bow cause distraction, which leads to a stress fracture or further deformation of affected femur. Oh et al. found that mechanical analysis identified significantly increased diffuse stress concentrated on the anterolateral portion of the femoral shaft throughout the whole length, in the patients with anterolateral femoral bowing deformity [27]. Oh et al. also reported that bone scintigraphy in patients with a preclinical stress fracture of the bowed femur showed mild diffuse uptake (not focal uptake) in the lateral cortex throughout the femur shaft [27, 28]. And Koh et al. reported that atypical femur lesions were clustered at the region of maximal tensile loading [29]. As such, we speculated that excessive lateral femoral bow may increase tensile stress on the lateral femoral cortex and that increased femoral bow may contribute to femoral cortical thickness to some extent.

We found no significant difference in medial cortical thickness between AFSF group and control group. However, we found that the lateral cortical thickness of the AFSF group was significantly larger than that of the control group. The generalized cortical thickness (sum of the medial and lateral cortical thicknesses) of AFSF group was also greater than control group. We believe that this finding is related to the increased lateral cortical thickness in AFSF group. Concordant result has been reported by Giusti et al., who analyzed the patients with subtrochanteric femoral shaft fractures who showed atypical radiologic features such as transverse or short oblique and noncomminuted fractures in an area of thickened cortices with unicortical beaking [3]. The femoral cortical thickness of the patients with radiologic atypia was significantly greater than that of patients without radiologic atypia. Besides, the ASBMR task force also adopted a general increase in cortical thickness of femur as one of the minor criteria for diagnosing AFFs [6]. Recently, Mahjoub et al. demonstrated the presence of AFF was associated with thicker lateral and medial bone cortices at the level of the lesser trochanter (*P* = 0.0137 and *P* = 0.0001) and 50 mm below the lesser trochanter (*P* = 0.0010 and *P* = 0.0006) [30]. On the other hand, Sasaki et al. reported no significant difference in femoral lateral cortical thickness measured at the thickest portion between the low-energy femoral shaft fractures group (BP users) and the control group (age and gender matched nonfracture patients) (7.3 ± 1.4 mm versus 7.3 ± 1.2 mm, *P* = 0.424) [17]. However, the differences in magnification and femur size were not adjusted in Sasaki's study.

When it comes to measuring cortical thickness, previous few studies analyzed thicknesses of the medial and lateral cortex separately while most studies only analyzed total cortical thickness of the femur [3, 16, 18, 19]. We considered it reasonable to measure medial and lateral cortices separately based on biomechanical studies demonstrating the distribution of femoral strain changed under loading—the tensile strain increased in the lateral side and the compressive strain increased in the medial side [31]. Different result in cortical thickening between lateral (tensile) side and medial (compressive) side demonstrated in our study is noteworthy, which is also dissimilar to general expectation of cortical thinning by the pathophysiology of age-related bone loss and Wolff's law [32] and warrants further biomechanical investigations.

In a regression analysis, anterolateral femoral bow and outer cortical diameter and lateral cortical thickness at the thickest portion of the lateral femoral cortex were factors significantly determining the presence of atypical radiologic features of postmenopausal femoral shaft fractures. No regression model showed a significant influence of medial cortical thickness on the presence of atypical radiologic features of postmenopausal femoral shaft fractures, and we assume geometric differences of the femoral diaphysis affect the pathogenesis of AFSFs to some extent. This observation is supported by recent studies which demonstrated AFFs were associated altered unbalanced tensile strain pattern in the lateral femoral shaft during walking [33], an underlying hip geometry [9, 34], or larger tibiofemoral angle [17].

When investigating the relation between femoral bow and cortical thickness, we found increased lateral femoral bow was positively correlated with lateral CTI (*R* = 0.378, *P* = 0.002). So far, only a few studies have investigated the relation between them in AFSFs. Contrary to our findings, Maratt et al. reported no significant correlation between femoral bow and cortical thickness, but only 2% of the sample was of Asian race in this study [35]. Previously Sasaki et al. tried to evaluate the femoral bow and cortical thickness of AFSFs in Asian females; however, the case series did not demonstrate meaningful association between them [17]. Regarding the relations between femoral bow and cortical thickness of AFSFs, it would be worthwhile to look into possible contributors to tensional bone failure as biomechanical demonstration in animal models clearly indicates that AFSF is caused by tensile failures of the lateral cortex of the femoral shaft [15, 36]. This plausible mechanism causing AFSFs can be a result of changes to the maturity of crosslinks formed by enzymatic processes or the accumulation of advanced glycation end-products. The latter have been experimentally shown to occur in association with high doses of BP in animal models [37]. To our knowledge, this is the first report demonstrating an association between an increased anterolateral femoral bow and lateral cortical thickening in patients with AFSFs; and this radiographic feature appears to be an important parameter causing AFSFs combined with BP therapy.

The strength of this study is the study design and relatively correct measurement of radiographic parameters. We documented that females with AFSFs, when compared to matched controls with a similar location of femoral fracture, demonstrated different femoral geometric parameters compatible with higher baseline tensile forces on the anterolateral femoral bone, the site of predilection for AFSFs [38]. To minimize possible confounding factors, only femoral shaft fractures excluding proximal and distal femoral fractures were selected as control group. A comparison of AFSF group with control group with proximal or distal femoral fractures could have been prone to selection bias. Additionally, it is important to match ethnicity, sex, age, and height because femur geometric parameters are affected by these parameters [39, 40]. So we tried to identify specific femoral characteristics of patients with AFSFs by comparing to age-, gender-, and ethnicity-matched controls with TFSF. But we still have to confess that height was not considered for this study because of lack of information during chart review.

The main limitation of our study is related to the small sample size and study design. We appreciate that case-control studies do have limitations, which include their nonrandomized nature leading to possible biases (such as selection and confounding) and the potential for misclassification. However, we feel this study design is appropriate for the following reasons. AFSF are rare events (as exemplified by our small sample size) thus justifying a case-control design. And, to decrease the risk of misclassification, our case definition based on the ASBMR task force was very rigorous with two independent observers with professional orthopedic knowledge. Second, there was lack of classification of proximal femoral geometry using Dorr type during our analysis. Nash and Harris reported clinically significant differences in cortical thickness at 10 cm below the lesser trochanter, among Dorr proximal femur morphology [41]. As proximal femoral geometry may be related to the cortical thickness in the shaft, a difference in cortical thickness among each Dorr type may be a confounding factor during our analysis. Lastly, we used radiographs as an imaging modality. It is possible that bone scan or MRI would be a much more sensitive imaging modality and would identify more lesions. The clinical utility of these advanced imaging modalities as a screening study for an uncommon condition would be limited, however, due to the increased expense and patient time required. Similarly, femoral bow was measured on plain radiograph instead of using computed tomography (CT). Measuring femoral bow on reconstructed CT is the more accurate way than measuring on the plain radiograph because femoral bow seen on the plain radiography can be affected by patient's position and femoral torsion [42]. However, we had not taken CT scan as routine procedure on femoral shaft fractures to avoid unnecessary radiation exposure. And while finding out exact femoral geometric parameters of AFSF is beyond the scope of this study, we recognize that femoral geometric parameters, especially lateral femoral bow on AP plane, documented in our participants are slightly different from those demonstrated in the Asian population, known to be at higher risk of AFF [43]. And Asian females have been known to have smaller proximal femurs [44], which made it difficult for us to compare femoral geometry parameters in this study to other ethnicities in direct numerical figures. To overcome these measuring biases, we used the same imaging modality for measuring radiographic parameters, all radiographs were taken in the same institution, and all radiographs were reviewed twice by two independent observers. And excellent intraobserver and interobserver reliability of the measurements were noted in this study as mentioned earlier. And we think the difference in femoral geometric parameters with previous studies was partly due to using a technique different than others [17].

In conclusion, despite some limitations, our study has shown that AFSFs were associated with an increased anterolateral femoral bow and a thicker lateral cortex of femurs in a sample of female BP users, and correlation analysis showed that the lateral femoral bow is solely related to lateral CTI.

## Figures and Tables

**Table 1:** Comparison of radiographic measurement data between AFSF group and control group.

	AFSF	Control	*P* value^*∗*^
Femoral bow			
AP radiograph (degrees)	12.39 ± 5.387	3.97 ± 3.62	<0.0001
Lateral radiograph (degrees)	15.71 ± 5.62	10.72 ± 4.61	0.002
Medial CTI	0.236 ± 0.06	0.240 ± 0.05	0.773
Lateral CTI	0.323 ± 0.04	0.267 ± 0.04	<0.0001
CTI	0.558 ± 0.07	0.507 ± 0.08	0.009
Adjusted lateral cortical thickness (mm)	10.5 ± 1.4	8.1 ± 1.3	<0.0001

^*∗*^Age corrected value.

All data were presented as mean ± standard deviation.

AFSF: atypical femoral shaft fracture, AP: anteroposterior, and CTI: cortical thickness index.

**Table 2:** Odds ratios of each variable after adjusting for age and body mass index.

	*P* value	OR	95% CI of OR	
			Lower	Upper
Lateral (coronal) femoral bow	<0.0001	1.546	1.234	1.935
Anterior (sagittal) femoral bow	0.005	1.220	1.064	1.400
Total cortical thickness	0.002	1.411	1.131	1.759
Lateral cortical thickness	<0.0001	3.234	1.779	5.878
Medical cortical thickness	0.521	1.090	0.837	1.421
Lateral cortical thickness index^*∗*^	<0.0001	1.526 × 10^14^	3.005 × 10^6^	7.746 × 10^21^
Medical cortical thickness index	0.769	0.238	0.000	3376.113
Adjusted lateral cortical thickness^†^	<0.0001	4.187	2.018	8.688
Adjusted medial cortical thickness^†^	0.478	1.114	0.826	1.5032

^*∗*^High OR for lateral cortical thickness index was caused by statistically significant difference of index between AFSF group and control group. Instead, we analyzed the OR between two groups by using adjusted cortical thickness^†^.

^†^Adjusted lateral and medial cortical thicknesses were calculated as the lateral and medial cortical thickness index multiplied by the mean femoral diameter of the AFSF group and the control group, respectively.

CI: confidence interval. OR: odds ratio.
